# How has the COVID‐19 pandemic changed patient care and the practice of medical physics in an academic environment?

**DOI:** 10.1002/acm2.13104

**Published:** 2020-11-29

**Authors:** Mary Beth Allen, Brian Wang, David Jordan, Michael Mills

**Affiliations:** ^1^ University of Louisville Louisville KY USA; ^2^ Radiation Oncology Physics Yale University New Haven CT USA; ^3^ Professor and Director of Radiation Oncology Physics Yale University New Haven CT USA; ^4^ Department of Radiology University Hospitals Cleveland Medical Center Cleveland OH USA; ^5^ Present address: Health Policy Specialist for the Commonwealth of Kentucky KY USA

The onset of the COVID‐19 pandemic has transformed all aspects of society. It has changed the way we interact as communities, socially and professionally. Although some of these changes may be temporary, other changes have added unanticipated benefits to many organizations and will likely remain integrated into our society in the future. The field of medical physics is no exception, as our turf has experienced significant changes as a result of modifications in the work flow, upgrades in safety protocols, and continued focus on quality patient care and satisfaction. Among the ways medical physics has been impacted is the movement toward working from home, the increasing reliance on technology for education and scholarship, implications for regulation, and the need to re‐examine revenue models of the past and apply it to current practice.

The movement toward working from home (WFH) is one of the most significant changes to our society. For many industries, WFH has so far been found to be beneficial for both the employer and the employee (although data on the long term are not yet available). For the employer, WFH has allowed significant cost savings in infrastructure and business costs without sacrificing employee productivity. For employees, WFH has resulted in savings of work expense and time that both support work–life balance and employee satisfaction. The field of medical physics has been no exception to these dynamics as its historic embrace of emerging technology has arguably made this transition to WFH smoother. This has resulted in the wide adoption of WFH policy having the potential to transform the field.

At Yale New Haven Hospital, it was encouraged during the early months of the onset of Covid‐19 lockdown policies by the Human Resource (HR) department to transition to WFH in all fields where appropriate, which included physics and dosimetry. Approximately 70% of more than 40 physicists and dosimetrists practiced WFH in the early months, with some employees preferring working on‐site while others preferred the WFH setting. The barriers to WFH for the former group included technological challenges such as unreliable internet, tedious VPN servers, or the challenge of duplicating the three‐monitor setup available on‐site in a home office. Some of these challenges experienced in our field are well described in a recent article published by NPR, “Time to Ditch Those Awful Zoom Calls, CEOs Say,” as many other fields experienced many of the same challenges. https://www.npr.org/2020/10/14/923428794/from‐the‐folks‐who‐brought‐you‐boring‐meetings‐ceos‐want‐to‐ditch‐sterile‐zoom‐c Other workflow challenges that were exposed include the benefit of in‐person interactions that allow the immediate resolution of questions and complexities associated with patient care. Being unable to reach a provider has implications for quality of care, patient satisfaction, and profitability. Also, provider interaction in a care facility in both the formal and informal sense has driven much of the in‐person workflow that can be difficult to duplicate in a WFH format. In fact, some argue that in‐person interaction is critical to the field in order to demonstrate the value imaging physics providers must reinforce to administrators. Also, in‐person interaction may facilitate a more efficient workflow, relationships, trust, and respect from colleagues. Furthermore, WFH was noted by many to place providers in a relaxed environment with more distractions acting as a barrier to productivity as compared to a normally regimented and disciplined at‐work environment. Finally, given the circumstances of this transition, it was found that regular rounding and interaction with frontline healthcare workers provided important emotional support to overwhelmed essential medical staff at work.

Despite the challenges of WFH to our field, it is notable that it also provided some key advantages. For example, large hospital systems such as Yale New Haven Health with multiple care centers were able to shift staff much more easily within the system to better accommodate patient volume fluctuations at each location. Also, there are several factors that illustrate the impact of WFH on employee satisfaction, including the elimination of commute times, decreasing of work expenses, and supporting a better work–life balance. There are employees who have found WFH to negatively impact work–life balance, notably, those who have school‐age children who cannot attend school. However, once the initial investment of technology and infrastructure in the workplace akin to the hospital setting was established for many providers in the home, the result for most was greater flexibility to accomplish work tasks. These responsibilities might involve an unplanned task during traditionally off‐hours or a hybrid WFH and in‐person work arrangement that sustains the response to the pandemic and provides benefits to both the professional and the organization. Although many consulting groups have been very cooperative with their employees, allowing them to work more or less as they are able with considerable support, the treatment toward these professionals by hospital systems has sometimes been less accommodating. In some situations, consultants found themselves "locked out" of their client hospitals and healthcare facilities as non‐employees; in many cases, visiting professionals were subject to being classified as nonessential vendor guests, especially during the early days of the pandemic. Furthermore, the cost of traditional business interaction, which sometimes involved travel for in‐person meetings with partners, vendors, and other associates, as well as in‐person continued education and conferences for professional organizations were replaced with lower cost virtual interaction.

Regardless of the benefit or challenge WFH created, it is important to note that the transition was abrupt, unplanned, and had important consequences. When WFH was implemented in UH Cleveland Medical Center (UHCMC), there was an accumulation of a backlog of all of the annual equipment testing that would have come due during that time but which was deferred under regulatory allowances. When in‐person work resumed in May, staff found themselves catching up on equipment testing while simultaneously catching up on other internal projects. Furthermore, new disinfection protocols decreased patient throughput significantly, which hindered necessary and preventative maintenance and evaluations of equipment.

In addition to patient care, teaching and scholarship are critical aspects of the field of radiation physics. The most renowned institutions with the greatest reputations for clinical care also tend to be teaching institutions. Training the next generation of medical physicists along with promoting continued research and scholarship are of equal concern and are complementary to providing quality clinical care. Teaching and scholarship have historically depended on in‐person lectures and hands‐on care setting‐based instruction. Modifying traditional approaches using remote learning technology has been an adjustment, as many of the methods that work effectively for in‐person instruction were found to not have the same effect over a web conference. As many education providers have been forced to adjust to virtual instruction in a myriad of fields, the scholarship in virtual teaching is ongoing. It likely will transform in the future, but in the short term, the transition has not been easy or highly effective for the teacher or the student. Several models were implemented during this period, from the traditional didactic and in‐person approach to the virtual format. At UHCMC, there was a transition to a hybrid model at the beginning of September in order to allow the new first‐year radiology residents’ better opportunities to get to know the faculty in‐person. Remaining residents were asked to join conferences remotely in order to facilitate physical distancing. As UHCMC has taken the same approach to all of other educational and training responsibilities that depend on traditional lecture formats, there is no viable replacement for hands‐on training virtually or remotely.

The continuation of scholarship also depends on the continued publication of science. Because scholarship related to COVID‐19 was in huge demand, JACMP publisher Wiley initially expressed concern in their ability to manage the throughput of unrelated articles. Although the JACMP was expected to publish fewer articles in the short term, by the time of publication of the June issue, it was apparent that this journal would be able to keep up the throughput of articles into Early View publication. Beginning in the June issue, JACMP began to publish more articles per issue. As of early October, we have largely accomplished the catch‐up, and the most recent issue, September, contained 34 articles. Despite abrupt and systemic changes in workflow, there was no notable consequence on the submission of articles, the length of review, and the time from submission to publication. As most of this work is computer processing of words and images, the entire process including reviews, acceptance, and post‐production can be accomplished easily in a WFH setting. The JACMP is continuing to see an increase in submissions, acceptances, and articles published similar to years past, and has apparently not been adversely affected by the COVID‐19 pandemic.

Regulation is an important component of the healthcare field. Complying with the various government and professional regulatory bodies through inspections, credentialing, interviews, and audits remains an important source of the trustworthiness of radiation physics that contributes to its credibility as a field. The credentialing process usually depends on the inspection of facilities and records, interactions with established professionals, and other often intense and in‐person interactions. A recent site inspection at Yale New Haven Hospital was conducted virtually and was much less involved than a traditional, hands‐on inspection. Other regulatory actions were simply postponed to be conducted a later date. This has concerning implications for the future credibility of many healthcare specialties, as virtual inspections, as they are currently being practiced, are not equivalent, and it is unclear how regulatory practices will be impacted in the long term.

The pandemic has had a considerable financial impact on the entire economy and healthcare has been no exception. The Yale New Haven hospital network lost hundreds of millions of dollars relative to budget for the current fiscal year; this was approximately 8% of the total annual budget. Consequently, almost all capital projects are on hold and approval for new job positions is extremely difficult, if not impossible. Under such a challenging financial climate, all physics QA device replacements have been postponed. University Hospitals of Cleveland was forced to close several small facilities within the health system with chronically low imaging volumes, which have been kept open in the past to support patient access by expanding geographically. It is unclear as to whether all facilities will reopen, which has important implications for the accessibility of care in the future. As hospital systems have been forced to reprioritize and reorganize, it is unclear how many professions will be impacted.

In summary, COVID‐19 has brought many changes to the practice of radiation physics and society. Although many of these changes have been temporary, it is unclear how many will remain integrated into our daily practice and our lives as they represent an improvement in quality, a savings in cost, or another benefit that we may have never otherwise considered. This pandemic has allowed us the opportunity to reevaluate the way we practice, the way we add value to our field, the way we promote our importance, and the way we adapt using technology in a field that is arguably more comfortable and reliant on cutting edge technological innovation than most fields including those within medicine. We should also recognize the importance of asserting our value in an increasingly competitive climate with hospital systems with fewer resources and increasing demands to reduce overhead in order to remain profitable in a drastically changed post‐pandemic world.

